# Are We Afraid of Different Categories of Stimuli in Identical Ways? Evidence from Skin Conductance Responses

**DOI:** 10.1371/journal.pone.0073165

**Published:** 2013-09-11

**Authors:** Tengteng Tan, Han Li, Yingying Wang, Jiongjiong Yang

**Affiliations:** Department of Psychology, Peking University, Beijing, China; The University of Queensland, Australia

## Abstract

Studies have shown that emotional pictures attract more attention than neutral pictures, and pictures of living stimuli have similar advantage in driving attention (vs. nonliving). However, factors of emotion, category and picture context are usually mixed so that whether living and nonliving categories elicit different skin conductance (SC) responses, in both conscious and unconscious conditions, remains to be clarified. In this study, participants were presented with negative and neutral pictures denoting different living and nonliving concepts in conscious (Experiments 1 and 2) and unconscious conditions (40ms, Experiment 3) when their SC responses were measured. The picture context was manipulated in Experiments 2 and 3 as half including human-related information. In three experiments, the emotional levels of different categories were matched in different and identical cohorts of participants. The results showed that living pictures in a negative, high-arousing dimension elicited stronger SC responses than nonliving pictures. When nonhuman animals and inanimate objects were compared, the increased SC responses to animals was obtained only for negative pictures without human contexts in the conscious condition, but regardless of human context in the unconscious condition. These results suggested that contextual information and level of conscious awareness are important to modulate the animate advantage in emotional processing.

## Introduction

In our daily life, we experience fear to different kinds of things or situations. We are afraid when we see snakes, scenes of mutilation and earthquake, and war films. The fearful feeling is usually associated with changes in the endocrine and autonomic nervous systems (e.g., skin conductance, startle reflex), and quick fight-or-flight behavioral strategies [1–3]. However, not all fearful stimuli are equivalent. Phobias are more common with respect to some stimulus categories than others. An influential evolutionary-oriented theory regarding the role of stimulus category/content is the preparedness model [4,5]. This model proposes that stimuli that signal a threat to human ancestors during evolution influence attention and emotional processing. Fear is more readily learned and resistant to extinction for stimuli that are related to survival threats to our evolutionary ancestors (e.g., snakes or spiders) than to threats that have only recently emerged in our cultural history (e.g., guns or motorcycles) [5,6].

The preparedness model has gained supports from various studies, which showed that living fear stimuli are more resistant to extinction (vs. electrical equipment [7]; vs. guns and rifles [8]), more likely to be overestimated in their occurrence with shock [6,9], and more likely to be attended to than nonliving fear stimuli [10]. For example, in a visual search paradigm, a fearful target (e.g., a snake or spider) out of a grid-pattern array of neutral distracters (e.g., flowers or mushrooms) was detected more quickly than a neutral target out of a grid-pattern array of fearful distracters [10]. When presented with pictures of spiders, flight accidents and mushrooms, subjects showed bias to spiders or accidents, accompanied with increased skin conductance (SC) responses [11]. When more contents of pictures are used, studies have also observed that different contents of stimuli elicited different SC responses, with the largest SC changes in response to animal/human attack, mutilation and erotic pictures [11,12]. For example, In Bradley et al. (2001) study [2], 72 pictures were selected to comprise 18 different contents (8 positive, 2 neutral and 8 negative). For the negative pictures, the largest SC changes were associated with pictures of human/animal attack and mutilations, and the SC changes for other contents (e.g., pollution, illness, loss, accidents and contamination) were smaller.

On the other hand, the negative pictures usually have the highest arousal rating scores and the arousal scores are highly correlated with the SC responses, whether the pictures are negative and positive (e.g., erotic pictures) [11–13]. Thus the difference in the SC responses to various contents could be at least partially explained by the difference in their ability to arouse the subjects. Furthermore, the pictures that elicit the strongest SC responses overall are related to humans and animals (e.g., human attack, mutilation), and nonliving objects (e.g., guns) are not designed as a separate type of category to compare with living category. Therefore, whether living and nonliving categories elicit different physiological responses remains to be determined.

Recent studies have emphasized the importance of contextual information in emotional facial processing [14,15]. Studies have found that emotional body contexts, natural scenes and emotional voices affect processing of facial expression. For example, subjects were asked to attend to a face and make judgments about facial expression. When body expression was matched to the facial expression, subjects made the decision more quickly and accurately [15]. When contextual information is fearful, it is processed quickly and does not require conscious awareness of emotional expression [16]. Similarly, we most likely feel that the scene of a car crash containing deadly body or mutilation is as threatening as a scene of a snake biting a person. In this situation, the difference between snake and car crash may diminish due to the presence of human context. Previous studies observed comparable emotional responses to living and nonliving objects in some conditions, e.g., pointed guns and pointed snakes had comparable SC responses during conditioning extinction [17]. This could be because guns with sounds are more likely to be associated with a threatening situation, particularly with extensive and consistent experience. Therefore, the existence of human-related information indicates that the situation is threatening, either because humans could elicit threatening reactions (e.g., a gunshot) or because humans are in danger (e.g., a car crash). But without the control of the picture context, the effects of emotion, category and context may be mixed.

Another interesting question is whether living and nonliving stimuli are processed differently under unconscious conditions. The SC responses could be detected when subjects attended to masked fearful faces [18] and emotional pictures [19] (but see [20]). For example, when emotional pictures were presented very quickly, their SC level was comparable to those were presented for a long duration [21]. It is hypothesized that living fear stimuli are automatically processed but nonliving fear stimuli may require elaborative processing [5], but supportive experimental evidence is rare. By using a backward masking paradigm, Ohman and Soares (1993) showed that pictures of masked snakes and spiders (30 ms) led to increased SC responses and were more resistant to extinction than masked mushrooms and flowers [18]. However, Flykt et al. (2007) showed that when the stimuli was directed toward the observer, masked guns and snakes (25 ms) had comparable SC responses indicated in fear conditioning [22]. Thus whether living stimuli are processed in a different way from nonliving stimuli under unconscious conditions needs further investigation.

In sum, it is necessary to dissociate the effects of emotion, context and category to clarify whether living or nonliving categories elicit different SC responses, in both conscious and unconscious conditions. In this study, participants were presented with living and nonliving pictures in emotional and neutral valences when their SC was assessed. In Experiment 1, living (humans and animals) and nonliving contents of pictures were presented. Pictures of high- and low-arousal types were used to dissociate the effects of valence and arousal on the SC responses. In Experiment 2, to further examine the effect of context on the SC responses, nonhuman animals and inanimate objects in negative and neutral valences were presented with their contexts manipulated, half including human-related information and half not. In Experiment 3, to examine the SC responses to different categories in the unconscious condition, pictures of animals and objects were presented in human or nonhuman contexts using a sandwich-masking paradigm. Each picture was presented for 40 ms with a forward and a backward mask to ensure participants were unaware of the presence of the picture. Emotional and semanitc detection tasks were employed to ensure that the participants unconsciously processed the pictures.

In all three experiments, by matching arousal (and valence) levels of different categories in a different and an identical cohort of participants, we could obtain category effect when their emotional features were matched. We hypothesized that effects of emotion, category and context interact to determine the SC responses under conscious and unconscious conditions. Based on previous findings, negative pictures elicit stronger SC responses than neutral pictures, and living things attract more attention [23] and have biological significance than nonliving things, it is possible that living negative pictures lead to the strongest SC responses than pictures in other conditions. In addition, the SC response could be influenced by contextual information [14,15]. Therefore, if the category interacts with context and awareness levels to influence the SC responses, an animate advantage should be shown for certain but not all conditions.

## Experiment 1

### Materials and Methods

#### Participants

Thirty-six right-handed healthy students at Peking University (22.36 ± 2.66 years old, 19 males) participated in the study. The participants had either normal or corrected-to-normal vision without any history of neurological or psychiatric disorders.

#### Ethics Statement

All participants were paid for their participation and provided written informed consent in accordance with procedures and protocols approved by the Institutional Review Board of the Department of Psychology, Peking University.

#### Materials

**Figure 1:**
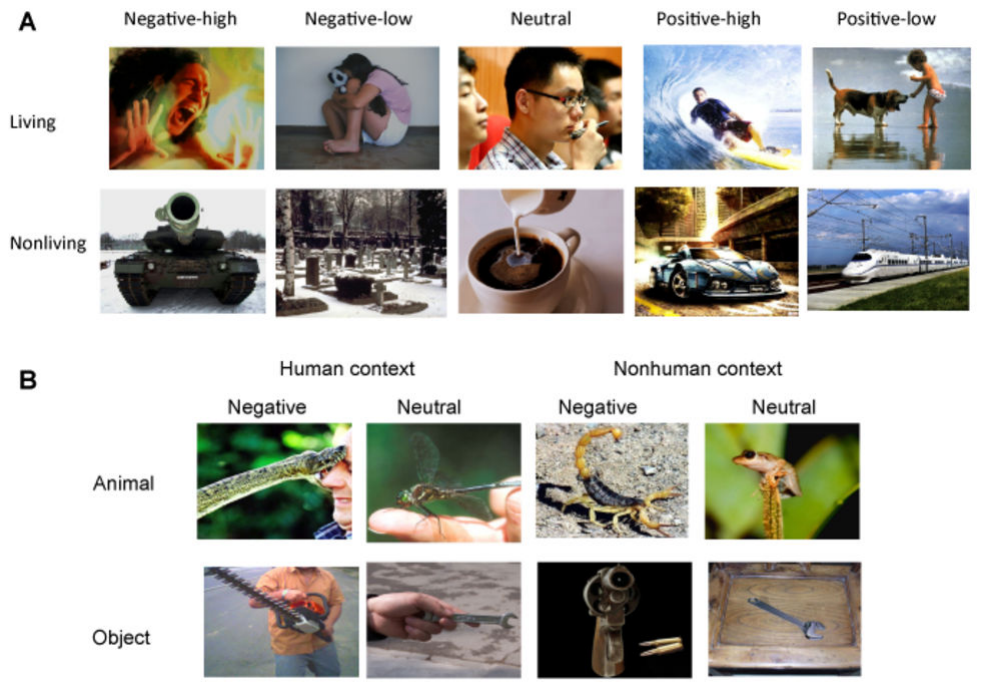
Stimulus example of Experiments 1 and 2.

Two within-subjects factors were included in Experiment 1: emotion (negative–low, negative–high, neutral, positive–low, and positive–high) and category (living and nonliving). The combination of the factors comprised 10 experimental conditions, e.g., negative living pictures with high arousal (i.e., negative–high living). The stimuli consisted of 80 color pictures (8 picture per condition) measuring 480 × 360 pixels each. Pictures containing humans or animals were defined as ‘living’ pictures. Other pictures containing only objects or scenes were defined as ‘nonliving’ pictures (Figure 1a). The number of human pictures was around 5-8 for the living pictures in five emotional conditions, and that of animal pictures was around 0-3. The backgrounds of all the pictures did not include human-related information.

The 80 pictures were selected based on their rating scores of valence, arousal, familiarity and complexity provided by a separate group of subjects (Text S1, Table S1). There were no significant differences in age or gender (ps > .2) between the two groups. Twenty-three of the 80 pictures were selected from the International Affective Picture Series (IAPS) [24] (numbers 1604, 1930, 2210, 2331, 2370, 2655, 3022, 4660, 5450, 5950, 6020, 6610, 7031, 7325, 8370, 8501, 8540, 9001, 9041, 9046, 9210, 9270, 9594). The mean valence scores of these pictures were comparable between our ratings and those in the IAPS database (4.80 vs. 5.38, p = .32), and the arousal scores were higher for our ratings than for those in the IAPS database (5.64 vs. 4.82, p < .05). Nevertheless, there were significant correlations for the rating scores between the two rating groups (valence pearson r = .84, arousal r = .63, both ps < .001).

For the selected 80 pictures, as study designed, negative pictures had the lowest valence scores (F(4,15) = 168.75, p < .001, η^2^ = .92), and high-arousal pictures had the highest arousal scores (F(4,15) = 68.36, p < .001, η^2^ = .82). There were no significant differences between high and low arousal picture in either negative or positive valence (p = 1.00). There were also no significant differences between negative and positive pictures in either high or low arousal level (p = 1.00). Importantly, living and nonliving pictures were rated with comparable scores for valence (F(1,15) = .23, p = .637, η^2^ = .02) and arousal (F(1,15) = 1.47, p = .244, η^2^ = .09), with no significant interactions between emotion and category (Fs < 1.0, p > .80) (Table 1). These data suggested that the affective features of the pictures are optimally matched across the categories.

**Table 1:** Valence and arousal scores in Experiment 1.

		NH		NL		Neutral		PH		PL	
		Living	NonL	Living	NonL	Living	NonL	Living	NonL	Living	NonL
Valence	Mean	2.63	2.66	2.89	2.98	5.06	5.16	6.85	6.92	7.09	7.12
	SD	0.82	0.99	0.50	0.82	0.74	0.84	1.09	1.08	0.87	1.08
Arousal	Mean	7.12	6.92	5.15	4.97	3.98	3.84	6.85	6.94	4.00	3.88
	SD	0.79	1.14	1.33	1.21	0.87	1.10	0.91	0.72	1.36	0.92

Note: NH: negative-high arousal; NL: negative-low arousal; PH: positive-high arousal; PL: positive-low arousal; NonL: nonliving.

#### SC procedure

The subjects participated in a preparation and a task phase. They sat in a chair in a small, sound-attenuated and dimly lit room with a temperature of approximately 20°C. During the preparation phase, electrodes were then placed on each participant’s left hand. To obtain reliable recordings and allow the equilibration of the hydration level and sodium at the interface between the skin and electrode paste, the participants were instructed to sit quietly for approximately 10 min.

During the task phase, the participants were informed that they should attend to each picture the entire time when it was presented on the screen. They were instructed to remain still and avoid any head movements, deep breathing, coughing or yawning. Each picture was presented for 3 s, and the average inter-picture interval was 6 s (range 5–7 s [21]). To ensure that the participants attended to the pictures, they were also instructed to rate the arousal level for each picture on a scale from one to five (least to most arousing). All pictures were randomly separated into four blocks with a 2-min break between each block. Each block consisted of 12 s of fixation at the beginning and a series of 18 pictures in a pseudorandom order. No more than three pictures with the identical affective level were presented consecutively. The participants were presented practice trials before the formal task phase to be familiar with the experimental procedure.

#### SC apparatus and data analysis

The SC signal was acquired with a Galvanic skin response system from Contact Precision Instruments (London, UK). The utility of this SC system has been verified [22,25]. During the experiment, 8-mm-diameter silver/silver chloride (Ag/AgCl) skin conductance electrodes filled with .05 M NaCl paste were placed adjacently on the medial phalanx of the index and middle fingers of the left hand. The SC data were sampled at 100 Hz and analyzed using PSYLAB8 data analysis software.

The data analysis was based on previous studies [2,12,13,22,26]. All data were transformed into change scores by subtracting the activity during the 1 s interval preceding each picture onset. The SC magnitude was used as the parameter to reflect the peak activity to a certain kind of stimuli. The SC amplitude was first scored as the maximum response that occurred between 1 and 4 s after the picture appeared [2,12,13]. Then to reduce skew and kurtosis distribution, a logarithmic transformation (log[SCR+1]) was performed. All data (including zero responses) were included in the analysis and the results of the SC magnitude were reported. Data from six participants were excluded because of an absence of SC responses across the experiment (i.e., the percentage of SC responses was lower than 10%). In average participants responded to 31% (range 11%-53%) of the stimuli, and the mean percentage for each condition was around 22%-38%. A repeated measures of ANOVA was conducted using the SPSS software (SPSS Inc., Chicago, IL, USA) with emotion and category as the within-subjects factors (two-tailed, p < .05). All ANOVA tests were reported with the Greenhouse–Geisser correction, and post-hoc pairwise comparisons were subjected to a Bonferroni correction.

### Results

#### Rating task performance

The arousal scores were significantly correlated with the arousal scores rated by the rating group (r = .85, p < .001). The emotional effect was significant (F(4,116) = 45.46, p < .001, η^2^ = .62). Both negative and positive pictures had higher arousal scores than neutral pictures, ps < .001, but there were no significant differences between negative-high and positive-high pictures, negative-low and positive-low pictures, ps > .20. Living pictures had comparable arousal scores to nonliving pictures (F(1,29) = .65, p = .429, η^2^ = .02). The interaction between emotion and category was marginally significant (F(4,116) = 2.33, p = .061, η^2^ = .08). This was because the living pictures were rated higher than nonliving pictures only for the negative–low pictures (p = .012) but not for the other types of pictures (ps > .20). These data confirmed the results of stimulus preparation and indicated that living and nonliving pictures evoked comparable arousal levels for the participants who were measured by the SC response.

#### SC responses

**Figure 2:**
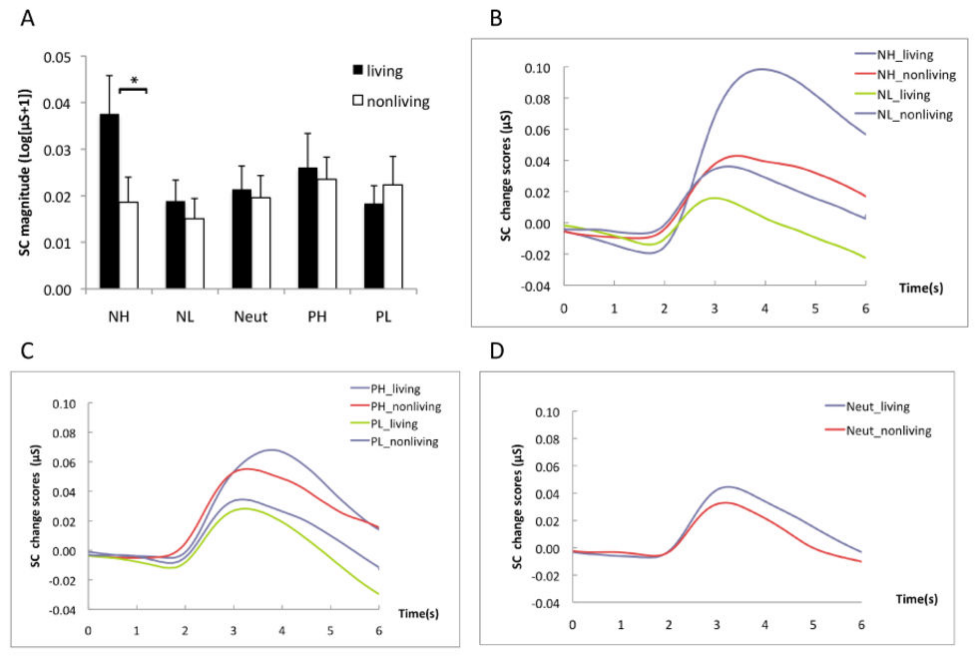
The results of Experiment 1. Living pictures showed stronger responses in SC magnitude than nonliving pictures in the negative-high condition (A). The average SC change waveforms are illustrated for negative (B), positive (C) and neutral (D) pictures by condition. Error bars represent standard errors of the mean.

The ANOVA showed significant main effects of emotion (F(4,116) = 4.92, p < .001, η^2^ = .14), and category (F(1,29) = 9.41, p = .005, η^2^ = .25). Negative–high and positive–high pictures elicited stronger SC responses than negative–low pictures (both ps < .04). The SC responses were comparable for negative-high and positive-high pictures (p = 1.00), negative-low and positive-low pictures (p = .59). There was also a significant interaction between emotion and category (F(4,116) = 3.00, p = .021, η^2^ = .09), which showed that living pictures elicited stronger responses than nonliving pictures in the negative–high condition (p = .002) but not in other conditions (ps > .30) (Figure 2a). The interaction also indicated that the emotion effect was significant only for living pictures, which showed that the SC responses were stronger for negative–high pictures than for other types of pictures (p < .05). Figure 2 (b–d) illustrated the SC changes as a function of picture emotion and category. Living pictures produced stronger SC changes than nonliving pictures, particularly in the negative–high condition. These results indicated a significant influence of category on SC responses when the affective features were matched across categories. It is also notable that the peak amplitude was located approximately 3-5 s, particularly for the negative pictures. We also performed an analysis for a 1-5 s time window, and the results were similar to those above. To be more conformed to the SC wave changes in the Chinese population, we selected 1-5 s after the stimulus onset as the time window in Experiments 2 and 3.

## Experiment 2

### Materials and Methods

#### Participants

Forty-two right-handed healthy students at Peking University (20.90 ± 2.15 years old, 17 males) participated in the experiment. The participants had either normal or corrected-to-normal vision without a history of neurological or psychiatric disorders.

#### Ethics Statement

All participants were paid for their participation and provided written informed consent in accordance with procedures and protocols approved by the Institutional Review Board of the Department of Psychology, Peking University.

#### Stimuli

Three within-subjects factors were included: emotional valence (negative or neutral), category (nonhuman animals or inanimate objects) and context (with or without human parts). The context/background of half of the pictures included human or human-related information (e.g., hand, foot), and that of the other half did not. The combination of the three factors comprised eight experimental conditions as demonstrated in Figure 1b. The target stimuli comprised of 120 colorful, nameable pictures (15 per condition), and each measured 480 x 360 pixels. The pictures represented 27 concepts: seven negative (e.g., spider, snake, etc) and six neutral nonhuman animal concepts (e.g., cow, sheep, etc); seven negative (e.g., gun, syringe, etc) and seven neutral inanimate object concepts (e.g., hammer, wrench, etc). Most concepts were presented in both contexts with and without humans (or human parts). The orientation of the stimuli was matched across conditions.

Picture selection was based on the results from another group of valence and arousal ratings. For the 120 selected pictures, the negative pictures were rated significantly lower in valence (F(1,20) = 238.75, p < .001, η^2^ = .92) and higher in arousal (F(1,20) = 85.66, p < .001, η^2^ = .81) than the neutral pictures. Compared to pictures without a human context, those with a human context were rated lower in valence (F(1,20) = 108.05, p < .001, η^2^=. 84), and higher in arousal (F(1,20) = 49.98, p < .001, η^2^ = .71). Similar to Experiment 1, animals and inanimate objects had comparable scores in both valence (F(1,20) = .01, p = .945, η^2^ = .01) and arousal ratings (F(1,20) = 1.05, p = .317, η^2^ = .05). There were no significant interactions related to category (Fs < .60, p > .40) (Table 2). These data confirmed that the affective features were matched across the categories.

**Table 2:** Valence and arousal scores in Experiments 2 and 3.

			Human context				Nonhuman context			
			Negative		Neutral		Negative		Neutral	
Experiment 2			Animal	Object	Animal	Object	Animal	Object	Animal	Object
Valence		Mean	2.40	2.46	4.73	4.57	3.42	3.46	4.95	4.96
		SD	0.90	0.61	1.07	0.46	1.20	0.76	1.21	0.43
Arousal		Mean	7.09	7.04	4.53	4.37	6.17	5.98	4.04	3.83
		SD	1.39	1.27	1.38	1.52	1.35	1.20	1.44	1.56
Experiment 3										
Valence	Mean		3.01	2.80	4.87	4.81	3.57	3.58	5.06	4.89
	SD		0.80	0.46	1.07	0.49	1.08	0.76	1.14	0.48
Arousal	Mean		6.63	6.61	4.42	4.29	5.89	5.83	3.96	3.94
	SD		1.19	1.35	1.25	1.32	1.14	1.19	1.26	1.44

#### Procedure

The procedure was identical to Experiment 1 except for the following: (1) each picture was presented for 6 s and the average inter-picture interval was 12 s (range 9–15 s) and (2) the participants rated the level of arousal for each picture during the interval on a scale of 1-7. All the pictures were randomly separated into four blocks with a 2-min break between each block. Each block consisted of 12 s of fixation at the start and a series of 30 pictures in a pseudorandom order. No more than three pictures with an identical emotional level were presented consecutively.

#### Data analysis

Data analysis was identical to Experiment 1, except that the SC response was scored as the maximum response occurring between 1 and 5 s after the stimulus onset [11,19], based on the results of Experiment 1. Note that we also performed the analysis for 1-4 s time window, and the results were similar to the current ones. In average participants responded to 31% (range 13%-49%) of the stimuli, and the mean percentage for each condition was around 27%-38%. Data from five participants were excluded because of an absence of SC responses in the experiment. In addition to analyzing the SC responses for all pictures, we compared the SC responses for pictures of snakes and spiders (N = 12) vs. guns and chainsaws (N = 14) to explore the difference between living and nonliving fear stimuli that were commonly used in previous studies [27,28].

### Results

#### Rating task performance

There was a significant interaction among category, emotion and context (F(1,36) = 8.59, p = .006, η^2^ = .19). However, a further pairwise comparison showed that nonhuman animals were rated significantly more arousing than inanimate objects only for neutral valence (ps < .001) but not for negative valence. There was no significant categorical effect for negative pictures in both human context and nonhuman context (ps > .10). The living and nonliving fear pictures were also matched in arousal rating. Although pictures in human context were rated more arousal than those in nonhuman context (F(1,29) = 68.88, p < .001, η^2^ = .70), there was no significant category effect (F(1,29) = .02, p = .901, η^2^ = .001), or interaction between category and context (F(1,29) = .509, p = .481, η^2^ = .017).

#### SC responses

**Figure 3:**
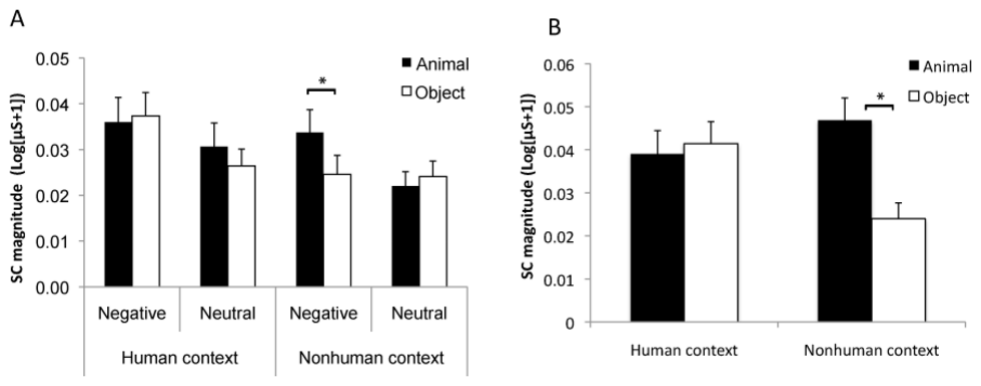
The results of Experiment 2. Nonhuman animals showed stronger responses in SC magnitude than inanimate objects only for negative pictures in the context without a human (A). The animate advantage in SC responses was also shown in nonhuman contexts when pictures of snakes and spiders were compared to those of guns and chainsaws (B). Error bars represent standard errors of the mean.

The ANOVA of category × emotion × context showed a significant contextual (F(1,36) = 6.20, p = .018, η^2^ = .15), and emotional effect (F(1,36) = 10.52, p = .003, η^2^ = .23) because negative pictures (vs. neutral) and pictures with human contexts (vs. without human contexts) elicited stronger SC responses. Notably, there was a significant interaction among the three factors (F(1,36) = 6.16, p = .018, η^2^ = .15). Further pairwise comparison showed that the SC responses were higher for nonhuman animals than for inanimate objects only in the negative condition without human contexts (p = .049) but not in the other conditions (ps > .20) (Figure 3a). The interaction also indicated that the emotional effect was significant for animal pictures regardless of the context, and for inanimate objects only in human contexts (ps < .02).

To compare the living and nonliving fear stimuli, there were no significant main effects of context (p = .37) and category (p = .07), but the interaction between category and context was significant (F(1,36) = 4.79, p = .035, η^2^= .12). The pairwise comparison showed that living pictures increased the SC response more than nonliving pictures in the nonhuman context condition (p = .011) but not in the human context condition (p = .739) (Figure 3b).

## Experiment 3

### Materials and Methods

#### Participants

Forty healthy students at Peking University (mean ± SD, 21.58 ± 1.69 years old, 19 males) participated in Experiment 3. The participants had either normal or corrected-to-normal vision without any history of neurological or psychiatric disorders.

#### Ethics Statement

All participants were paid for their participation and provided written informed consent in accordance with procedures and protocols approved by the Institutional Review Board of the Department of Psychology, Peking University.

#### Stimuli

The design was identical to that of Experiment 2. Most of the stimuli were the same as those used in Experiment 2, and some of them were replaced to be suitable for an unconscious presentation (e.g., lower contrast pictures). The pictures represented 29 concepts: eight negative and seven neutral nonhuman animal concepts; eight negative and six neutral inanimate object concepts. Most concepts were presented by pictures in contexts with and without humans (or human parts).

For the 120 selected pictures, the negative pictures were rated lower than neutral pictures in valence (F(1,20) = 229.34, p < .001, η^2^ = .92) and higher in arousal (F(1,20) = 104.42, p < .001, η^2^ = .84). Similar to that in Experiment 2, Pictures with a human context were rated lower in valence (F(1,20) = 52.90, p < .001, η^2^ =. 73) and higher in arousal (F(1,20) = 67.47, p < .001, η^2^ = .77) than those without a human context. Nonhuman animals and inanimate objects had comparable rating scores in valence (F(1,20) = .34, p = .565, η^2^ =. 02) and in arousal (F(1,20) = .20, p = .662, η^2^ = .01) (Table 2). No significant interactions related to the categories were observed (ps > .4).

#### Procedure

**Figure 4:**
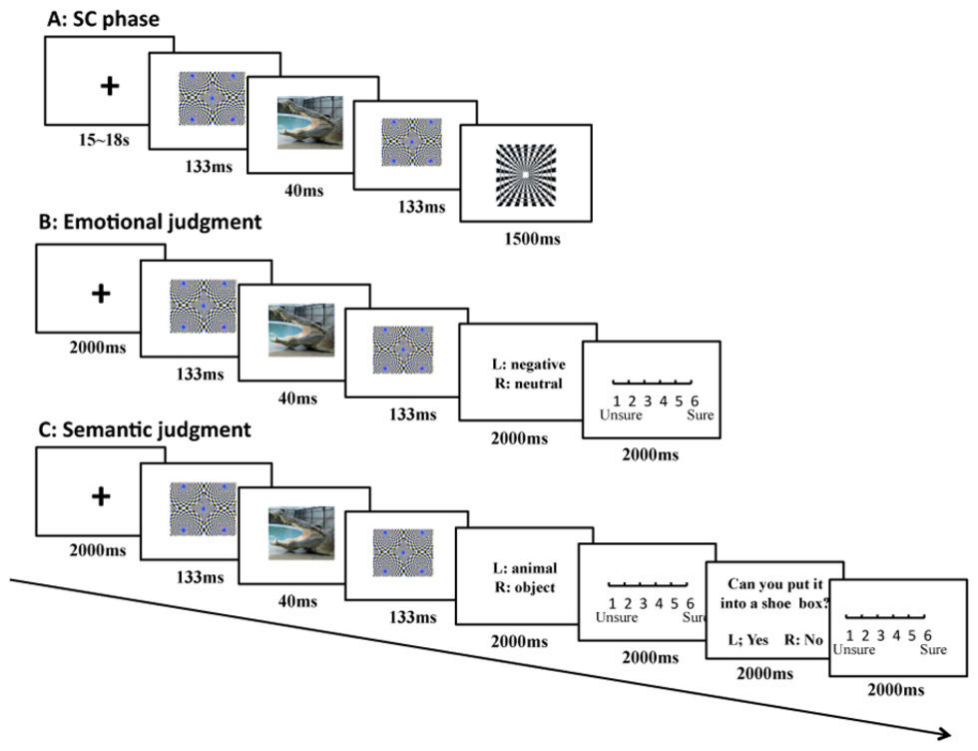
The procedure of Experiment 3. The sandwich-mask paradigm was used to present the pictures (A). The following emotional judgment (B) and semantic judgment (C) tasks were used to assess the participants’ levels of conscious awareness.

The participants performed the following tasks sequentially: the SC test phase, awareness detection phase (Figure 4) and emotional rating phase. The distance between the monitor and the participants’ eyes was approximately 60 cm. The refresh rate of the monitor was set at 75 Hz. During the SC phase, a sandwich mask paradigm was used. In each trial, a forward mask (133 ms), a stimulus picture (40 ms), and a backward mask (133 ms) were presented sequentially. Three abstract pictures were selected from the Internet to be used as masks. The masks were paired with the targets by color and pattern to effectively mask the target stimuli. The Identical masking picture was served as the forward and backward masks in each trial. Following the backward mask, a black-and-white square was presented randomly in one-quarter of the trials. The participants were asked to press a button within 1500 ms when they saw the square. The average inter-trial interval was 16 s (range 15–18 s). The pictures of each experimental condition were presented in blocks. The pictures in each block were pseudorandomly mixed to ensure that no more than three stimuli of the identical concept were consecutively presented. The order of the four blocks was counterbalanced across subjects.

After the test phase, the participants’ awareness was assessed by two tasks. The stimuli and sandwich-masking manipulations were identical to those in the SC procedure, but the stimuli were presented in different random orders. In the emotional judgment task, the participants were required to decide whether the content of the masked stimulus was negative or neutral and then rate their response confidence on a 6-point scale. In the category judgment task, the participants were required to make two judgements after each masked presentation: They first judged whether the content of the masked picture was a nonhuman animal or inanimate object, then judged whether the concept depicted by the picture could be placed in a shoe box based on its actual size. Each judgement was followed by a confidence rating to the response on a 6-point scale. Because we performed the semantic judgment several weeks after the SC measurement, only 22 participants were enrolled in this task.

To further verify our manipulation of the stimuli, the arousal rating from the SC participants were collected. After the emotional judgment task, the participants viewed the pictures and rated their arousal levels on a 9-point scale. The picture did not dissapear on the screen until they made the response.

#### Data analysis

Data analysis for the SC response was identical to that in Experiment 2. Data from four subjects were excluded because of extensive movement artifacts (one participant) and an absence of SC responses across the experiment (three participants). In average participants responded to 41% (range 19%-63%), and the mean percentage for each condition was around 31%-46%. Similar to Experiment 2, we compared the SC responses for the pictures of snakes, spiders and sharks (N = 10) vs. pictures of guns (N = 9) in addition to the analysis for all pictures. For the awareness assessment, the ROC scores were converted from the participants’ responses and confidence ratings, and the area under the ROC curves (A′) were computed for each participant and subsequently entered into SPSS for analysis. The A’ is used to estimate the ability to discriminate true positive and false positive trials (e.g., emotional vs. neutral pictures; animal vs. object pictures), and the A’ = .5 is a chance level of discrimination between them. The participants were considered as being unaware when their detection task performance was at chance level (i.e., the A′ values were not significantly different from chance level) in either emotional or category judgment task.

### Results

#### Rating task performance

The arousal ratings were significantly correlated with those of the other ratings group (r^2^ = .78, p < .001). The ANOVA revealed a significant effect of context (F(1,35) = 23.84, p < .001, η^2^ = .41). Negative pictures had higher arousal scores than neutral ones (F(1,35) = 153.07, p < .001, η^2^ = .81). Notably, there was no significant effect of category (F(1,35) = 1.11, p = .300, η^2^ = .03). All interactions were not significant (Fs < 1, ps > .30). For the comparison of living and nonliving fear stimuli, the ANOVA revealed a significant higher arousal scores for living fear stimuli than nonliving fear ones (F(1,35) = 16.05, p < .001, η^2^ = .31). There was also a significant effect of context on arousal, as pictures with human contexts had higher arousal scores than those without human contexts (F(1,35) = 12.00, p < .001, η^2^ = .26). The interaction between context and category was not significant (p = .529).

#### Awareness assessments

For the emotional judgment task, the A’ scores for all participants were not significantly different from chance level (.5), (t(35) = 1.60, p = .119), which suggested that the participants remain unconscious at the affective categorization level. For the semantic judgment task, only one of the 22 subjects performed above chance in the category judgment task (animal vs. object), (A’ = .71, p < .001). However, the subject failed to achieve above chance accuracy in the size judgment task (A’ = .56, p = .281), which suggested that his performance in the category judgment task might rely on a certain inference strategy rather than an analysis of the sensory input (e.g., I see a patch of red color. Although I’m not sure what it is, it is possibly an object because red animals are rarely observed). Therefore, this subject remained regarded as unconscious at the semantic categorization level. It is additionally notable that the SC results did not change when this subject’s data were excluded. The group analysis showed that the participants remained unconscious when they viewed the masked pictures for the category judgment (t(21) = .91, p = .372), and for the size judgment (t(21) = .57, p = .573).

#### SC responses

**Figure 5:**
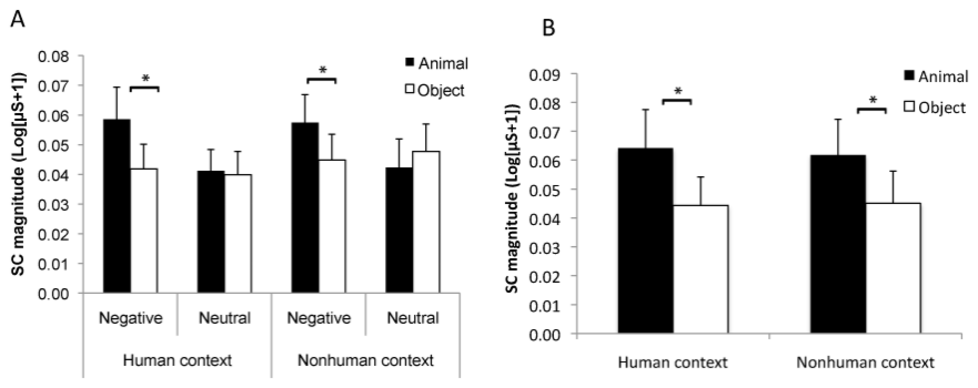
The results of Experiment 3. Nonhuman animals elicited stronger responses in SC magnitude than inanimate objects in the negative condition (A). The animate advantage in SC responses was also shown regardless of human context when the pictures of snakes, spiders and sharks were compared to those of guns (B). Error bars represent standard errors of the mean.

The ANOVA results showed a significant emotional effect. Negative pictures elicited higher SC responses than neutral pictures (F(1,35) = 6.51, p = .015, η^2^ = .16). The category effect was not significant (F(1,35) = 2.71, p = .109, η^2^ = .07), but the interaction between emotion and category was significant (F(1,35) = 6.17, p = .018, η^2^ = .15). A post-hoc analysis indicated that animal pictures elicited stronger SC responses than objects for negative pictures (p = .023) but not for neutral pictures (p = .786) (Figure 5a). The interaction also indicated that the emotional effect was significant for animal pictures regardless of context (ps < .05) but not for inanimate objects (ps > .50). The three-way interaction was not significant (F(1,35) = .01, p = .918, η^2^ = .01), suggesting that the animate advantage for negative picture was regardless of context.

We also compared the SC responses between living and nonliving fear stimuli. The ANOVA with context and category as factors showed a non-significant effect of context but a significant effect of category on SC response (F(1,35) = 6.32, p = .017, η^2^ = .15). The interaction between context and category was not significant (p = .92), which suggested that animals elicited stronger SC responses than objects whether the context included human information (Figure 5b). The ANOVA with context and category as factors showed no significant effect of context but a significant effect of category on the SC magnitude (F(1,35) = 6.32, p = .017, η^2^ = .15). The interactions between context and category were not significant (p > .80). However, because the stimuli were not well matched by the within-group arousal ratings (although after the SC measurement), we performed a regression analysis to explore the effect of arousal. The factors of arousal, category and context were entered into the regression using a stepwise approach. The results showed a significant effect of arousal rating (F(1,119) = 5.75, p = .018, model r = .21), and category (F(1,119) = 5.41, p = .022, model r = .22), when they were first entered the model. Adding category at step 2 (after arousal) and adding arousal at step 2 (after category) both significantly increased the R scores, ps < .05. Therefore, both arousal and category contribute to the SC responses in the unconscious condition.

## General Discussion

The objective of this study was to investigate whether different categories of emotional pictures modulate the SC responses in conscious and unconscious conditions. The emotional features were controlled across categories by between and within groups. The results showed that living pictures in a negative, high-arousing dimension elicited stronger SC responses than nonliving pictures. When nonhuman animals and inanimate objects were compared, the animate advantage was obtained only for negative pictures without human contexts in the conscious condition, but regardless of human context in the unconscious condition. These results indicated that the category, emotional feature, and human context interact to modulate the emotional processing in both conscious and unconscious conditions.

### Increased SC responses to negative living pictures

One of the main findings of our study was that negative living pictures elicited stronger SC responses than negative nonliving pictures. The category effect on emotional stimuli has been reported in previous studies using physiological measures, which showed that pictures of animal/human attacks and mutilation elicited stronger SC changes than other types of negative pictures [2,12]. However, because these pictures were also highly arousing, and the SC response was highly correlated with ranked arousal rating, it is unclear whether different arousal levels cause the effect of content/category on emotional processing, or whether the category effect is independent of affective dimensions. Our study obtained the animate advantage when the emotional features were matched across categories. We initially balanced the valence and arousal levels of the different categories in the three experiments. Weakly arousing pictures in both categories (living or nonliving) were also included and all pictures were matched on valence, arousal, complexity, and familiarity in Experiment 1. In addition, we obtained SC and rating data from the identical cohort. The participants whose SC was measured also rated the arousal level of each picture and showed similar arousal scores for different categories in three experiments. Although the arousal rating scores in Experiment 2 showed a significant interaction among category, emotion and context, the category effect was significant only for the neutral pictures but not for the negative pictures, which was different from the pattern of SC responses.

Across the three experiments, we observed a significant animate advantage in SC response only for the negative pictures, but not for the neutral or positive pictures. In addition, the results of Experiment 1 showed that only high-arousing negative pictures had an animate advantage. These results suggested that animate advantage occurs to threatening stimuli, which are negative and highly arousing. By contrast, some studies using visual search paradigms observed that there was an animate advantage for pictures of neutral (vs. flowers and mushrooms in [29]) and positive animals (vs. plants in [28]). When neutral animals were learned as CS+, the subjects rated them as more negative and preferential attention [30]. When the participants were asked to detect a difference between two rapidly alternating scenes that were identical except for a change to one object, they were faster and more accurate at detecting changes in animals than in other objects [31]. Our previous study using the eye-tracking technique [32] also observed that an animate advantage occurred for both negative and neutral pictures. These results suggested that humans and animals attract stronger attention than inanimate objects regardless of their valence levels.

Nevertheless, our results were not contradictory to these results because they may reflect different aspects of responses to emotional stimuli. Visual search paradigms and eye-tracking techniques are related to attentional allocation, whereas the SC response is an autonomic response to emotional stimuli. The SC response is not only related to attentional allocation [2,33–35], but also associated with action preparation to avoid threatening situations [12]. This is consistent with the role of the sympathetic system in arousing and engaging behavior in response to significant events. Quickly identifying the danger from animate stimuli and adopting an effective strategy is important for human survival. Although there is comparable attention allocation for fearful and neutral animals and for fearful animals and objects [27,36,37], the participants’ SC responses may be different and reflect stronger autonomic reactions and action preparations to negative biological pictures [38]. Our study suggested that only threatening, but not neutral stimuli, show a strong animate advantage in physiological responses.

### Comparable SC responses for negative pictures with human contexts

Living and nonliving pictures may also differ in their contextual information, which influences the SC responses [23,39]. Therefore, we conducted Experiment 2 by adding contextual information as a factor to determine whether the picture context influences animate advantages in emotional processing. The results of Experiment 2 illustrated a significant interaction among context, emotion and category, and showed that pictures of animals and objects elicited comparable SC responses when they included human contexts. When pictures did not include human contexts, animal pictures elicited stronger SC responses than inanimate objects. The emotional features were matched by between- and within-group controls, showing that negative animals were rated aroused at the similar level to negative objects, in both human and nonhuman contexts. Although there was category effect for neutral pictures in within-group controls, this pattern was different from the SC responses because neutral pictures did not show a significant category effect in the SC responses.

To comply with previous studies, we directly compared the SC responses of living and nonliving fear pictures. The snakes and spiders were rated similarly to guns and chainsaws in both human and nonhuman contexts, but their SC responses were stronger only for nonhuman context. It suggested that the animate advantage do not apply to all nonhuman animal pictures, and highlighted the effect of human context in fear processing. Previous studies have also found that faces and objects that are congruent with their contexts were identified better and faster [15,40,41]. In this study, we manipulated contextual information as human-related or not and central stimuli as animals and nonliving objects in different affective levels. Contextual information constrains what we expect to see and where we look [15]. Participants can use contextual human information to make inferences regarding the intentions of conspecifics [42,43] and about the seriousness of the situation. For example, people may differently attend and react to a gun on a table vs. a gun handled by a human hand because the latter indicates the potential presence of a threatening situation and a quick emotional response is required. When negative inanimate objects were placed in the context with humans or human-related information, they were also attended to and led to strong SC responses at similar levels to negative animals.

These results also helped us to understand the inconsistent findings in previous studies [17,32]. To be supportive evidence, we previously observed that in images with a negative valence, animals and objects with a human context had a comparable number of gaze fixations and durations in an eye-tracking experiment [32]. It suggested that category and picture context interact to influence attentional allocation in emotional processing. At the same time, the system begins to prepare for action which was indicated by the pattern of SC responses. This was consistent with the view that emotion is organized by appetitive or defensive systems that involve a transition from attention to action [44]. The affective responses serve different functions (e.g., attention, preparation for action, and social communication), and varies according to the specific emotional context.

On the other hand, fear of living and nonliving origins may be related to different cognitive and brain mechanisms. Some researchers hypothesized that the fear of evolutionary-related animals is automatic and prepared, whereas the fear of recently emerged objects may depend on their contexts [36,45,46]. It is important to learn the contexts of these objects, thus making it possible to prepare ahead of a threatening situation. Therefore, regions related to object action [46] and top-down regulation, e.g., the prefrontal cortex [36], may be involved in processing nonliving fear stimuli. Studies have shown that the amygdala activity is highly associated with SC responses in humans and monkeys [19,25,47]. After the left amygdala was impaired, the correlation between the SC responses and arousal scores decreased [19]. In addition, subcortical regions, anterior cingulate cortex and prefrontal regions are related to SC responses [35,48]. These regions are involved in contextual evaluation [49,50] and emotional regulation [51]. Further studies are needed to clarify whether the activity of these regions are modulated by contextual information when living and nonliving categories of fearful stimuli are used.

### Increased SC responses to unconsciously negative animal pictures

Our study further showed that in the unconscious condition, nonhuman animals elicited a stronger SC response than inanimate objects (Experiment 3), which supported the preparedness theory that processing of phylogenetic fear stimuli does not rely on conscious awareness. Different from Experiment 2, the animate advantage occurred even when pictures included human contexts. The present results indicated that compared to negative inanimate objects, negative nonhuman animals have a priority for attendance and action preparation in the unconscious condition. This quick detection mechanism is important for human beings because they could rely on limited information to prepare in action even when they are not aware of the presence of threatening stimuli. It is important to ensure that the participants process the pictures unconsciously. We applied the sandwich-masking paradigm, in which the mask appeared before and after the target picture to allow for an optimal masking effect. Moreover, we tested the participants’ level of awareness by both emotional and semantic judgment tasks. The participants remained unconscious to the masked pictures because they performed at chance level in both tasks. The rationale to add a semantic judgment was that semantic categorization may precede affective evaluation of visual scenes [52]. Some studies did not observe an animate advantage in the unconscious condition. It is possible by extensive learning (e.g., conditioning), the culturally-related threat could elicit a strong automatic response [22], particularly when the weapon was combined with a noise burst.

Unconscious processing relies on the semantic difference between animal and object pictures rather than their affective and perceptual differences. First, we obtained an animate advantage in the unconscious condition when the affective levels were matched across categories in both between- and within-group ratings. Second, semantic categorization was faster and more accurate than affective processing even with short exposures [52] (for reviews, see [55,56]). Semantic information could be extracted when a scene was presented for 20-40 ms [53,54]. and the level of semantic categorization (e.g., snake detection, gender detection, etc.) did not change the pattern [52,57]. Third, category distinctions may be more important than perceptual features in unconscious conditions. In Sebastiani et al.’s (2011) study [57], phobic patients only showed increased SC responses for spiders (but not for crab and squirrel) in the unconscious condition. Removing color information of emotional pictures did not change the late positive potentials regardless of whether the presentation duration was 24 ms or 6 s [58].

In addition, previous studies suggested that emotional and semantic features are processed earlier than contextual neutral information. For example, an eye-tracking study observed that during the initial fixations, the animate advantage occurred for negative pictures regardless of their contexts [32]. Subjects did not have sufficient time to process the contextual information under the unconscious condition due to quickly presentation and limited cognitive resources. This led to comparable animate advantage for pictures with and without human-related contexts.

It should be noted that the unconscious processing of emotional pictures is not equivalent to an explicit identification. Participants may unconsciously process emotional stimuli without access to their affective evaluations. For example, although snakes and spiders were preferentially attended to in a visual search task, negative evaluations were not automatically elicited during the processing [31]. In addition, unconscious processing does not include a complete pattern of autonomic responses to threatening stimuli. The heart rate did not show significant changes during subliminal presentations [57,59], which may be related to the absence of prefrontal activation. In our study, the living fear pictures elicited a stronger SC response than nonliving fear pictures. Although the participants consciously rated the living fear pictures as more arousing than the nonliving fear ones, this result does not mean that they felt the identical arousal levels in the unconscious condition. Nevertheless, we performed a regression analysis and showed that both arousal and category accounted for the animate advantage for living fear pictures.

### SC responses for emotional pictures

A number of studies have reported that emotional stimuli, including both negative and positive pictures, elicited stronger SC responses [13]. In this study, we also obtained the same conclusion for negative pictures in three experiments. The SC responses of animals and objects were then compared when their emotional features were matched. Furthermore, we included pictures depicting large sets of animals and objects in Experiments 2 and 3, rather than only several types of concepts. It ensured that the conclusion could be applied to more generalized pictures. Different from Experiments 2 and 3, we included both negative and positive pictures in Experiment 1. The results showed that the overall SC responses were comparable for negative-high and positive-high pictures, suggesting that positive pictures elicit strong physiological responses as well, although the category difference was not significant.

The interactions between category and emotion were also manifested as a significant emotional effect for living but not for nonliving pictures (except in Experiment 2, in which the inanimate objects with human contexts showed significant emotional effects). This result was consistent with the fact that many previous studies showing an emotional effect in SC responses used living stimuli as material [2,12,39,60]. Note that within the living category in Experiment 1, negative–high pictures elicited stronger SC responses than those in neutral or positive dimensions, but positive–high pictures did not produce a similar pattern. This result might be because only two erotic pictures were included in the positive-high condition. It is no doubt that erotic pictures elicit stronger SC responses than other positive pictures [2,12,39], but including other positive-high pictures (e.g., extreme sports) could better match with other types of pictures, and reflect a general pattern of the SC responses to positive-high pictures.

### Clinical significance and limitation

Among specific phobias, animal phobia has the highest prevalence [61,62]. Why specific phobias are preferentially observed to threatening living stimuli? Our results suggested that there are specific mechansims to living fear stimuli (vs. nonliving counterparts). Compared to nonliving fear stimuli, living fear stimuli attract more attention, elicit stronger physiological reactions and preparatory actions. So we may be afraid of negative animals (vs. objects) when they are presented without human context or without conscious awareness, although they are rated at similar affective levels. On the other hand, some people suffer from posttraumatic stress disorder (PTSD) that is characteristic of the overwhelming terror resulting from certain trauma, especially in people who underwent life-threatening events [63]. Our result highlighted the importance of human contexts in the nature of phobias to inanimate objects. The significance for the mechanism is that threatening events related to nonliving fear stimuli occur more frequently in modern society. Therefore, people prepare to respond not only to living fear stimuli but also to any stimulus that might be labeled as dangerous to human beings [45]. PTSD patients are possibly afraid of not the object itself, but the object that is located in a certain situation.

Our study has some limitations. One limitation was that negative and neutral pictures in Experiments 2 and 3 differed in both valence and arousal levels, so their SC difference (i.e., emotioanl effect) could be explained by both valence and arousal. As for the animate advantage, only SC magnitude were adopted as main variables in this study. Other SC measurements [26,64] and psychophysiological parameters (e.g., startle reflex) [2,13] could be additionally used to get a more comprehensive view for the category effect in processing emotional stimuli. In addition, the model-based methods have been used to analyze the SC response data in recent years [26,65]. Further studies could use causal models to see if other latent factors modulate the SC responses in the current design.

## Conclusions

In conclusion, our study showed that when the valence and arousal levels were matched across categories, the animate advantage in the SC responses was prominent only for negative pictures, in both conscious and unconscious conditions. But this advantage disappeared when the human context was included in the conscious condition. These results highlighted that the contextual information and level of conscious awareness are important factors to modulate the animate advantage in emotional processing. This study helps us understand the etiology of some emotional disorders, and highlights the effect of stimulus category on modulating emotional responses, including subjective ratings, autonomic responses, and brain activations.

## Supporting Information

Text S1 Supplementary material for Experiment 1. It included stimulus preparation, results and discussion of familiarity and complexity ratings, and references.
(DOC)

Table S1 Familiarity and complexity scores in Experiment 1. (DOC)
