# Supplementary material for: Are We Afraid of Different Categories of Stimuli in Identical Ways? Evidence from Skin Conductance Responses
Source: PLoS One. 2013 Sep 11;8(9):e73165. doi: 10.1371/journal.pone.0073165 (PMC3770652; doi:10.1371/journal.pone.0073165)
Supplement: Text S1 — It included stimulus preparation, results and discussion of familiarity and complexity ratings, and references. (DOC) [file pone.0073165.s001.doc]

# Supplementary Materials

## Stimulus preparation of Experiment 1

***Emotional rating.*** To measure the emotional features of the pictures, 240 pictures (96 unpleasant, 96 pleasant and 48 neutral) were selected from the IAPS and the Internet. Pictures of various animals, humans, objects and scenes were included. Participants rated valence (1 = most unpleasant to 9 = most pleasant) and arousal (1 = least arousing to 9 = most arousing) levels of each stimulus.

***Familiarity and complexity ratings.*** To reduce the influence of other factors in the SC responses, we controlled for picture complexity and familiarity levels across the categories. For the familiarity rating, the participants were asked to rate how often they encountered or thought of the presented stimulus in their daily life (1 = least familiar to 7 = most familiar). For the complexity rating, the participants were asked to rate how many details were included and how many changes were in the contours of the presented stimulus (1 = least complex to 7 = most complex). The participants initially completed the familiarity rating and then the complexity rating after the emotional rating task. The pictures were presented in a pseudorandom order and the order of four blocks was counterbalanced across participants.

## Familiarity and complexity ratings in Experiment 1

The results of familiarity ratings showed a significant interaction between emotion and category (F(4, 15) = 5.91, p = .002, η^2^ = .28), because the negative pictures were less familiar than neutral or positive pictures (ps < .01), but the neutral and positive pictures were similar in their familiarity rating (p = 1). In addition, when compared with the nonliving pictures, living pictures were more familiar in negative–high level but were less familiar in positive levels (ps < .02). There was also a significant emotional effect (F(4, 15) = 24.92, p < .001, η^2^ = .66). The negative-high pictures were less familiar than negative-low and positive-high pictures, but positive-high and positive-low pictures had comparable familiarity rating scores (p = 1.00). There was significant emotional effect for the complexity ratings (F(4, 15) = 10.65, p < .001, η^2^ = .42), because positive–high pictures were more complex than other picture types (ps < .03). There were no significant difference between negative-high and negative-low pictures, negative-low and positive-low pictures (ps > .50). The category effect and the interaction were not significant (Fs < 2, p > .20) (Table S1).

Previous studies have suggested that SC responses could be significantly influenced by stimulus arousal level [1,2]. Stimulus features such as novelty and familiarity also influence the SC responses [3]. The feeling of familiarity was related to SC responses that are stem from autonomic arousal [4]. Our results showed that negative pictures were more familiar than neutral and positive pictures, whereas the living pictures were less familiar than nonliving pictures. Living and nonliving pictures had similar complexity rating scores. Although there were familiarity rating differences between categories, the discrepancies between the SC data and the rating data suggested that the differences could not account for the SC response patterns. The results of the familiarity rating (living > nonliving pictures and negative < neutral pictures) were different from the SC results (living > nonliving picture and negative > neutral pictures). The results suggested that the familiarity should not be the main factor to determine the SC response patterns.

Our results showed that the stimulus complexity was matched across categories, thus category difference could not be explained by complexity. In addition, previous studies suggested that the SC responses do not critically rely on perceptual features such as brightness, complexity or color, even when presented very briefly [1,5-6]. For example, Junghofer et al. (2001) showed that discrimination between low and high arousing pictures was independent of formal pictorial properties (e.g., color, brightness, spatial frequency, and complexity) [5]. Removing color information had no effect on the affective modulation of the neural activity, regardless of exposure duration [6]. Effects of perceptual composition may appear earlier in time (150–250ms), and longer effects on brain activity and SC responses are minimal [7].

## References

1. Bradley MM, Codispoti M, Cuthbert BN, Lang PJ (2001) Emotion and motivation I: Defensive and appetitive reactions in picture processing. Emotion 1: 276-298.

2. Lang PJ, Greenwald MK, Bradley MM, Hamm AO (1993) Looking at pictures - affective, facial, visceral, and behavioral reactions. Psychophysiology 30: 261-273.

3. Critchley HD (2002) Electrodermal responses: What happens in the brain. Neuroscientist 8: 132-142.

4. Morris AL, Cleary AM, Still ML (2008) The role of autonomic arousal in feelings of familiarity. Consciousness and Cognition 17: 1378-1385.

5. Junghofer M, Bradley MM, Elbert TR, Lang PJ (2001) Fleeting images: A new look at early emotion discrimination. Psychophysiology 38: 175-178.

6. Codispoti M, Mazzetti M, Bradley MM (2009) Unmasking emotion: Exposure duration and emotional engagement. Psychophysiology 46: 731-738.

7. Lang PJ, Bradley MM (2010) Emotion and the motivational brain. Biological Psychology 84: 437-450.
