# Supplementary material for: Are We Afraid of Different Categories of Stimuli in Identical Ways? Evidence from Skin Conductance Responses
Source: PLoS One. 2013 Sep 11;8(9):e73165. doi: 10.1371/journal.pone.0073165 (PMC3770652; doi:10.1371/journal.pone.0073165)
Supplement: Table S1 — (DOC) [file pone.0073165.s002.doc]

Table S1 Familiarity and complexity scores in Experiment 1

|  |  | NH |  | NL |  | Neutral |  | PH |  | PL |  |
| --- | --- | --- | --- | --- | --- | --- | --- | --- | --- | --- | --- |
|  |  | Living | NonL | Living | NonL | Living | NonL | Living | NonL | Living | NonL |
| Familiarity | Mean | 3.72 | 3.25 | 4.47 | 4.48 | 5.62 | 5.77 | 4.93 | 6.19 | 5.16 | 5.88 |
|  | SD | 0.96 | 0.89 | 1.42 | 0.89 | 1.07 | 1.08 | 1.38 | 0.85 | 1.20 | 1.04 |
| Complexity | Mean | 4.38 | 4.76 | 4.18 | 4.42 | 4.41 | 4.52 | 5.35 | 5.85 | 4.31 | 4.81 |
|  | SD | 0.85 | 1.22 | 1.18 | 1.12 | 0.83 | 1.13 | 0.92 | 1.21 | 1.11 | 1.36 |

Note: NH: negative-high arousal; NL: negative-low arousal; PH: positive-high arousal; PL: positive-low arousal; NonL: nonliving.
